# Scientific autonomy in the structural bubble: from institutional bias to AI-mediated consensus

**DOI:** 10.3389/frma.2026.1766504

**Published:** 2026-04-07

**Authors:** Elias Rubenstein

**Affiliations:** Independent Researcher, Fort Lauderdale, FL, United States

**Keywords:** automation bias, citation bias, consensus infrastructures, epistemic governance, large language models, meta-research, metrics performativity, peer review

## Abstract

Most professional science is produced inside institutions whose survival depends on competitive funding, political legitimacy, and reputation management. Under these conditions, knowledge production does not unfold in a neutral space of ideas but within a structurally constrained environment—a *structural bubble*—in which only some questions, methods, and conclusions are likely to be funded, published, and disseminated at scale. This article models four interacting layers: (1) institutional funding and conflicts of interest; (2) publication systems, peer review, and metrics as selection and valuation mechanisms; (3) consensus infrastructures (policy-governed encyclopedic and secondary synthesis platforms) that stabilize dominant framings under non-scientific governance rules; and (4) *artificial intelligence (AI)*-mediated access systems that operate as recursive second-order filters by reproducing prestige-weighted patterns through citation bias, over-generalization, and automation bias. The manuscript's incremental contribution is to theorize how AI-mediated synthesis and discovery reshape epistemic authority and legitimacy *as workflow infrastructure* (not merely as efficiency tools), to make this mechanism explicit in a minimal formal sketch, and to specify safeguards (validation loops, traceability, and oversight) that condition when AI strengthens vs. weakens scientific autonomy. Governance implications are derived from social epistemology and the sociology of quantification rather than presented as normative add-ons, and boundary conditions are sharpened with explicit falsifiers. Finally, the paper proposes a minimal empirical program to quantify agenda alignment, consensus lock-in, and amplification effects in AI-mediated discovery, summarization, and gatekeeping.

## 1 Introduction: science within the structural bubble

In normative self-descriptions, modern science appears as a self-correcting enterprise driven by evidence, methodological rigor, and open criticism. Classic accounts such as Merton's analysis of the “normative structure of science” emphasize values like communalism, universalism, disinterestedness, and organized skepticism (Merton, 1973). In practice, however, most scientific work takes place in structured organizational and economic environments.

Researchers are typically employed by universities, public research institutes, hospitals, agencies, or corporate laboratories. These organizations compete for funds, prestige, and political influence. Their continued existence depends on being perceived as successful, reliable, and aligned with certain policy or market priorities. Under such conditions, the production of knowledge is shaped by constraints that extend beyond the internal logic of scientific method.

In this article, the term *structural bubble* denotes a semi-closed environment in which certain research trajectories are systematically more feasible than others because funding, institutional incentives, publication filters, and prestige hierarchies jointly constrain the set of questions that can be asked, the methods that are rewarded, and the conclusions that are likely to be disseminated at scale. This term is descriptive rather than accusatory: it does not presume intent, collusion, or misconduct. It names a recurrent constraint pattern that can be studied empirically as a set of incentives, selection effects, and visibility mechanisms.

### 1.1 Scientific autonomy (definition as used in this framework)

In this framework, *scientific autonomy* denotes the degree to which (i) research agendas remain diverse and are not narrowly constrained by agenda-bearing resource dependencies, (ii) epistemic authority tracks evidential and methodological appraisal rather than prestige proxies, and (iii) discovery and synthesis workflows remain resilient against visibility concentration and default-framing lock-in under platform and AI mediation. The manuscript uses *scientific autonomy* consistently in these three coupled dimensions: **agenda autonomy**, **epistemic autonomy**, and **workflow autonomy**.

**Agenda autonomy** denotes the diversity and independence of the question space from agenda-bearing resource constraints. **Epistemic autonomy** denotes the degree to which epistemic authority tracks evidential and methodological appraisal rather than prestige proxies (venue tier, affiliation, cumulative advantage). **Workflow autonomy** denotes the resilience of discovery and synthesis workflows against visibility concentration and default-framing lock-in under platform and AI mediation. In this framework, autonomy is operationally readable via measurable signatures such as agenda diversity, long-tail inclusion, attribution diversity, and the coupling between prestige proxies and surfacing probability.

### 1.2 Operationalization sketch (measurable proxies)

To avoid leaving the concept at the level of metaphor, the structural-bubble construct can be operationalized with measurable proxies that correspond to the layers modeled below. Examples include: (i) *funding concentration and agenda alignment* (share of third-party funds by source class; topic drift toward funder-priority language); (ii) *publication and evaluation pressure* (venue-tier dependence of career outcomes; citation-velocity thresholds in evaluation); (iii) *record visibility concentration* (top-*k* venue share; affiliation concentration among highly surfaced work); and (iv) *AI-mediated surfacing concentration* (retrieval/summarization diversity, attribution entropy, long-tail inclusion under matched queries). These proxies connect institutional dependence and quantification mechanisms to empirically testable outcomes (Porter, 1995; Espeland and Stevens, 2008).

The concept overlaps with three adjacent ideas while differing in scope and mechanism. First, Kuhn's account of *normal science* emphasizes paradigm-guided puzzle-solving and resistance to anomaly-driven change (Kuhn, 1962). The structural-bubble model agrees that paradigms stabilize research, but emphasizes organizational dependence, evaluation systems, and external legitimacy constraints as additional stabilizers. Second, the notion of an *epistemic bubble* describes situations where relevant voices are excluded largely through structural omission rather than active distrust (Nguyen, 2020). Structural bubbles include epistemic-bubble mechanisms but extend them by focusing on institutional survival constraints and prestige-coupled filtering. Third, the popular concept of the internet *filter bubble* describes algorithmic personalization that narrows exposure to information (Pariser, 2011). Structural bubbles can incorporate algorithmic filters (especially in AI-mediated discovery), but their primary mechanism is upstream: the coupling of research agendas and dissemination channels to resource, reputation, and policy constraints.

### 1.3 What is established vs. what is AI-specific in this manuscript

A core concern in the science-of-science literature is that funding, publication hierarchies, and evaluation systems shape research agendas and visibility. Those mechanisms are well documented and are not “new” here. The incremental claim of this manuscript concerns how *AI-mediated workflows* (discovery, synthesis, summarization, and sometimes review support) can reconfigure epistemic authority and legitimacy by (i) stabilizing default framings through consensus-oriented substrates, (ii) increasing concentration on prestige-weighted sources via retrieval and citation dynamics, and (iii) compressing uncertainty and boundary conditions through over-generalization.

**The central incremental claim is:** “*AI-mediated access can shift epistemic authority from evidence-weighted appraisal toward prestige-weighted surfacing unless constrained by traceable provenance and validation-loop governance.”*

Crucially, bubbles are not only restrictive; they can also be *protective*. Science needs demarcation and quality control to avoid dissolving into arbitrary pluralism, unreliable claims, and predatory inclusion. The problem is therefore not the existence of filters but their opacity, their automation, and their coupling to incentives that can lock in agenda alignment and prestige advantages. The critique in this paper is not a call to abolish demarcation; it is a call to make demarcation more transparent, criteria-driven, and less dependent on opaque prestige automation.

### 1.4 Summary of contributions and foundational mapping

To address the requirement for analytical discipline, Table 1 maps the core mechanisms discussed in this paper to foundational literature, the specific “AI delta” proposed as an incremental contribution, and the resulting measurable observables.

**Table 1:** Mapping of mechanisms, foundational literature, and the proposed AI-specific incremental contribution.

Mechanism	Foundational literature	Incremental claim (AI delta)	Operational signatures
Cumulative advantage	Merton (1973); Bourdieu (1975)	AI-mediated second-order filtering via prestige-weighted retrieval.	Increased concentration in citation distributions within LLM synthesis.
Metrics performativity	Porter (1995); Espeland and Stevens (2008)	Transformation of metric-driven signals into automated epistemic authority.	Correlation between venue-tier weights and surfacing probability in AI.
Consensus stabilization	Kuhn (1962); Aibar et al. (2015)	Recursive lock-in through consensus-oriented secondary substrates.	Framing stability and reduction of claim-cluster entropy in AI summaries.
Epistemic governance	Longino (1990); Malik and Terzidis (2025)	Safeguards (traceability, validation) as essential boundary conditions.	Divergence in diversity outcomes between retrieval-augmented generation (RAG) vs. black-box synthesis.

In this manuscript, *retrieval-augmented generation (RAG)* denotes AI-assisted synthesis constrained by explicit source retrieval and auditable provenance, in contrast to non-retrieval, black-box generation.

**This paper makes four main contributions**. First, it synthesizes empirical findings from meta-research on conflicts of interest, peer review, and evaluation into a unified multi-layer model of structural dependence in science. Second, it integrates scholarship on consensus-oriented knowledge infrastructures into this model and treats Wikipedia as a prominent instance of a broader class of policy-governed secondary synthesis platforms that can function as practical reference substrates in public-facing knowledge practices (with field- and task-dependent academic use), while remaining non-scientific in governance and sourcing constraints (Okoli et al., 2014; Mesgari et al., 2015). Third, it theorizes AI-mediated access as a workflow layer that can reshape epistemic authority and legitimacy, and it integrates workflow governance (validation loops, traceability, and oversight) as boundary conditions on those effects (Malik and Terzidis, 2025). Fourth, it proposes falsifiable hypotheses and a minimal empirical program to quantify consensus lock-in and amplification effects under AI-mediated scientific discovery and gatekeeping.

**Figure 1:**
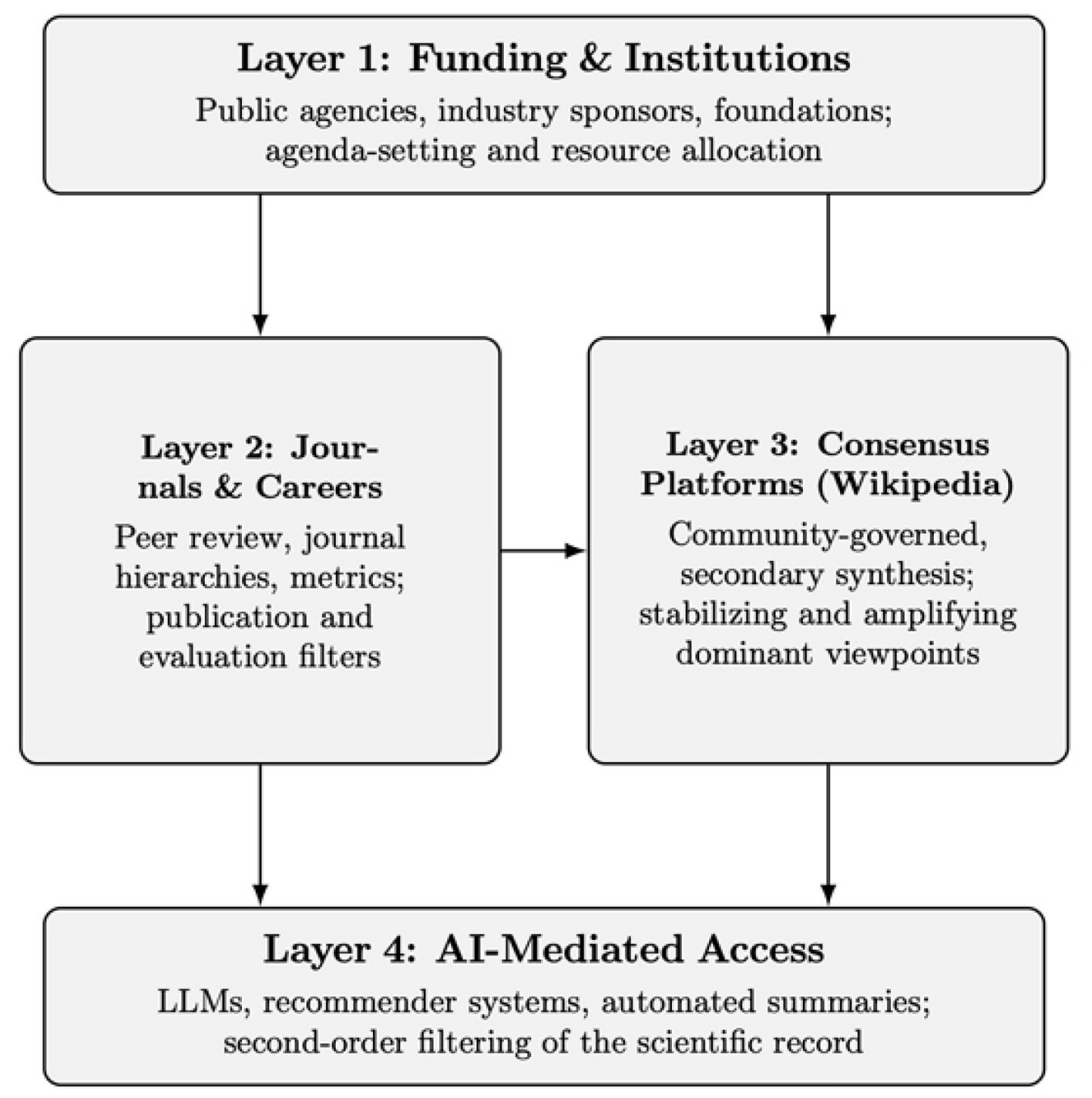
Structural decomposition of the layered model. Layer 1 (funding and institutions) shapes Layer 2 (publication and evaluation systems) and Layer 3 (consensus infrastructures); Layer 4 (AI-mediated access) can operate as a second-order filter. The figure is a conceptual schematic, not an empirical causal graph.

The focus throughout is not on individual misconduct but on system-level selection effects that can raise the cost of pursuing high-disruption, low-legitimacy, or cross-paradigm questions, particularly outside dominant institutional networks. Figure 1 provides a structural decomposition of the layers. The conditional recursive amplification pathway is described conceptually in the text as a feedback mechanism that depends on workflow design and governance (in particular traceability, validation loops, and oversight).

## 2 Institutional funding and financial conflicts of interest

### 2.1 Structural dependence on external funding

Across many research systems, competition for external funds has intensified and institutions rely more heavily on competitive grants, performance-linked allocation, and third-party contracts (OECD, 2024). The relevant point for this framework is an upstream feasibility filter: projects misaligned with dominant funder priorities are less likely to be initiated, shaping the distribution of pursued questions before later publication or evaluation filters.

### 2.2 Empirical evidence on financial bias

A substantial body of meta-research documents associations between industry sponsorship and more favorable outcomes for sponsors. Bekelman et al. (2003) report consistent links between industry funding and pro-industry results and conclusions. A Cochrane review by Lundh et al. (2017) similarly finds that manufacturer-sponsored drug and device studies more often report favorable efficacy results and conclusions than studies funded by other sources. Related discussions of bias and conflict-of-interest governance in biomedical research further reinforce the need to distinguish evidential appraisal from sponsor-aligned incentives (Bero, 2017).

### 2.3 Ideology, policy alignment, and institutional isomorphism

Not all conflicts of interest are financial. Public institutions depend on political support and on their symbolic role in national science and innovation systems, which couples leadership incentives to prevailing policy frames (e.g., growth, competitiveness, security, and industrial strategy).

Bourdieu argues that scientific fields allocate prestige and resources through internal hierarchies that are partly autonomous but also coupled to external power structures (Bourdieu, 1975). In practice, this can raise the career cost of positions that generate institutional friction, especially for early-career researchers.

Over time, these conditions foster internalized expectations about what is fundable, publishable, and publicly legible. These upstream pressures matter here because AI-mediated discovery and synthesis can inherit, compress, and amplify the resulting visibility patterns downstream.

#### 2.3.1 Scope note (proportionality)

Layers 1–3 specify upstream selection and visibility mechanisms that are well documented in the literature. The manuscript's incremental contribution concerns how AI-mediated workflows can inherit, compress, and reshape these patterns as a second-order filter, and how workflow safeguards can condition whether AI strengthens or weakens scientific autonomy.

## 3 The publication system and research evaluation as epistemic filters

If funding structures determine which projects can be undertaken, journals and peer review determine which results become part of the formal scientific record. Research evaluation systems then determine which of those results become *career-relevant*, and thereby which claims acquire *epistemic authority* under real institutional conditions. The publication-evaluation complex thus acts as a second major filter.

### 3.1 Peer review and social bias

Peer review is often framed as a central quality-control mechanism, yet reviewer judgments can be influenced by social and institutional factors. General reviews of the literature document multiple forms of peer-review bias across settings and disciplines (Lee et al., 2013; Haffar et al., 2019). (Wennerås and Wold 1997) and Wold found systematic bias patterns in reviewer scoring for grant applications. Tomkins et al. (2017) reported measurable differences under single- vs. double-blind review in a controlled conference setting.

### 3.2 Journal hierarchies, gatekeeping, and record formation

Publication is organized in a strong hierarchy, with a small group of highly selective, high-impact journals at the top and a long tail of lower-prestige outlets. Editors and reviewers in top-tier journals are embedded in well-funded institutions and networks. They face incentives to publish work aligned with fashionable topics and funder priorities, methodologically familiar to their readership, likely to generate citations, and less likely to produce reputational controversy.

Because hiring and promotion committees heavily weight journal prestige and citation counts, this structure reinforces itself. Researchers orient their work toward those expectations, and the journals in turn support the careers of researchers whose work fits dominant institutional and theoretical priors.

### 3.3 Metrics as performative mechanisms with epistemic consequences

Bibliometric indicators (impact factor, h-index, and citation counts) are often treated as neutral measures. In practice, they function as *performative instruments*: they reshape behavior and, under institutional dependence, they also reshape what counts as a legitimate contribution. Scholarship on quantification emphasizes that numerical indicators can become governance devices that translate trust and legitimacy into standardized signals (Porter, 1995; Espeland and Stevens, 2008). Mirowski (2011) describes how commercialization and intellectual property regimes have transformed scientific labor into market-oriented production. Under such conditions, metrics do not merely rank outputs; they can become default proxies of epistemic worth in careers, funding, and agenda setting.

For the structural-bubble model, the relevant step is to link metric performativity to *epistemic authority and legitimacy*. If evaluation systems reward certain outputs (publication venue tier, citation stock/velocity, and topical fashion), they indirectly shape which claims become widely cited, taught, and summarized. This pathway links evaluation to knowledge consolidation: the scientific record becomes selectively amplified in the direction of what is measurable, promotable, and institutionally legible.

### 3.4 Mechanism sketch: evaluation-weighted authority under AI-mediated access

Reviewer concerns about “explicit modeling” can be addressed with a minimal, operational mechanism that remains compatible with a conceptual article.

Let *A*(*s*) denote an *epistemic authority proxy* for a source *s*: the probability that *s* is (i) surfaced in discovery, (ii) cited, and (iii) treated as a default reference in synthesis workflows. Social epistemology treats authority as socially mediated trust in testimony and expertise, which is observable through adoption and deference patterns rather than reducible to truth alone (Goldman, 1999; Longino, 1990). Let *E*(*s*) denote evaluation weight (venue tier, citation stock/velocity, institutional prestige signals), and let *R*(*s*) denote the surfacing probability induced by the AI-mediated workflow (retrieval, ranking, summarization, and presentation).

A minimal mechanism is:


$$\begin{array}{l} A\left(s\right)\propto R\left(s\right)\cdot E\left(s\right),\\ R\left(s\right)=f\left(\text{retrieval rules},\text{training priors},\text{query},\text{safeguards}\right). \end{array}$$

Under weak safeguards, *f* tends to concentrate attention on high-*E*(*s*) sources (prestige-weighted retrieval and citation bias), and synthesis tends to compress uncertainty and boundary conditions (over-generalization). Under strong safeguards (traceability, validation loops, tier-aware diversification), *f* is constrained and *R*(*s*) can be forced to include methodologically accountable long-tail sources even when *E*(*s*) is low.

#### 3.4.1 Heuristic note and limits

This sketch is intentionally minimal: it is not a microfounded causal model of scientific authority, and it does not identify causal effects without additional assumptions. Its purpose is to make the dependency between evaluation-weighted signals and AI-mediated surfacing explicit and to generate testable predictions about concentration and diversity under different workflow designs. In empirical use, *A*(*s*), *E*(*s*), and *R*(*s*) are proxies that require field-specific operationalization and audit; functional forms need not be linear or stable across fields.

This yields testable predictions: holding topic and baseline search constant, AI-mediated workflows with weak safeguards increase concentration in *A*(*s*) (top-*k* share) and decrease attribution diversity; safeguards weaken or reverse this effect.

### 3.5 Demarcation and predatory inclusion under permeability

Any realistic account must also address the demarcation problem: communities require mechanisms that screen out claims that are not methodologically accountable. Making the system more permeable does not mean removing quality control; it means shifting quality control toward transparent criteria.

One risk of indiscriminate inclusion is predatory inclusion: low-quality outlets provide legitimacy signals while providing minimal peer review (Shen and Björk, 2015; Grudniewicz et al., 2019). Openness should increase permeability to unconventional work without collapsing demarcation. Visibility should therefore be conditional and tiered: claims should receive comparable standing only when they meet baseline scientific obligations, including explicit hypotheses, testable implications or a specified route to testability, transparent assumptions and methods, and a declared protocol for evaluation and potential falsification.

## 4 Consensus platforms as epistemic substrates

### 4.1 Wikipedia as one consensus platform, not scientific authority

Wikipedia is used here primarily as an illustrative case of a broader class of policy-governed secondary synthesis and reference platforms; the mechanism proposed in this manuscript does not depend on Wikipedia specifically. This layer concerns *consensus infrastructures*: platforms that stabilize public-facing framings through editorial rules (e.g., neutrality policies, sourcing requirements, and prohibitions on original research). Wikipedia is a prominent instance within this class and is widely used for quick background checks and initial familiarization, with variation across fields and tasks (Aibar et al., 2015; Jemielniak, 2019; Okoli et al., 2014). From a scientific perspective, treating such platforms as quasi-authoritative without explicit reflection on their governance and sourcing can introduce bias. Wikipedia is not a scientific journal; it is a community-edited, secondary compilation governed by rules such as “no original research” and “neutral point of view.”

Comparative work such as Giles' analysis in *Nature* suggests that, in relatively uncontroversial domains, Wikipedia can approach conventional encyclopedias in error rate (Giles, 2005). Problems concentrate in contested domains where incentives to shape narratives are high (Jemielniak, 2019). Systematic scholarship on Wikipedia content and quality further indicates that strengths and weaknesses are domain- and governance-dependent rather than uniform across topics (Mesgari et al., 2015). The implication for this model is conditional: consensus platforms can be convenient orientation tools, but they are structurally unsuited to act as arbiters in contested domains.

### 4.2 Structural biases and agenda-setting risks

Scholars of digital knowledge infrastructures have documented systematic biases in Wikipedia, including imbalances in topic coverage and concentration of influence on controversial pages (Jemielniak, 2019; Yasseri et al., 2012). Quantitative work has measured political slant in large article sets (Greenstein and Zhu, 2012). Case-based and quantitative work documents agenda-setting dynamics and *conflict-of-interest (COI)* patterns on contested pages (Jemielniak, 2019; Mesgari et al., 2015; Oppong, 2014; Beutler, 2020; Grabowski and Klein, 2023; Reagle and Koerner, 2020).

For scientific autonomy, the key point is structural: in contested domains, a consensus platform can be strategically shaped while remaining policy-compliant at the surface level. If such a platform (or analogous consensus infrastructure) becomes a privileged substrate in AI-mediated access, then those biases can travel downstream as default framings.

## 5 AI-mediated access as recursive second-order filtering

### 5.1 AI as workflow infrastructure, not generic amplification

Large language models (LLMs) increasingly mediate discovery, synthesis, summarization, and sometimes review support. Treating AI as a generic amplifier misses the workflow reality: epistemic effects depend on how AI is embedded in validation, traceability, and oversight. Methodological frameworks for AI-augmented synthesis explicitly articulate safeguards such as validation loops, provenance tracking, and human oversight (Malik and Terzidis, 2025). These safeguards are central boundary conditions for the model advanced here: AI-mediated access is most likely to intensify structural dependence where traceability is low, retrieval is prestige-weighted, and validation is weak.

#### 5.1.1 Process chain (second-order filtering as workflow dynamics)

In the layered model, second-order filtering can be read as a workflow process: upstream selection shapes the published record; the record is stabilized by evaluation and consensus infrastructures; AI-mediated retrieval and summarization then preferentially surface high-visibility patterns; those outputs are adopted as defaults in discovery, synthesis, and sometimes gatekeeping; and the resulting citations and attention feed back into evaluation signals. The mechanism is therefore conditional on workflow design: traceability, validation loops, and diversification constraints determine whether AI inherits and amplifies concentration or counteracts it.

### 5.2 Consensus substrates in training data and downstream surfacing

Documentation for large models such as GPT-3 lists Wikipedia as a component of training data (Brown et al., 2020). “Wikipedia in” does not imply “Wikipedia out” deterministically. The magnitude of consensus-substrate effects depends on corpus balance, fine-tuning, and whether deployed systems use retrieval augmentation that preferentially surfaces Wikipedia (or other consensus-oriented secondary sources).

Because LLMs do not expose internal source attributions by default, this process is opaque to end users. The combination of consensus-style synthesis and model fluency can produce *consensus lock-in*: once a framing dominates high-visibility summaries, it is disproportionately reinforced in AI-generated answers, which may then be used to justify the same framing elsewhere.

### 5.3 Inherited bias, citation bias, and over-generalization

Gallegos et al. (2024) survey evidence that LLMs learn and can amplify biases present in training data. In scientific contexts, this implies preferential reproduction of patterns from highly represented disciplines, regions, languages, and journals.

Algaba et al. (2025) found that LLM-generated references can mirror human citation patterns while exhibiting heightened citation bias toward already highly cited papers and prestigious venues. (Peters and Chin-Yee 2025) report systematic generalization bias in LLM summarization of scientific research, with limitations and boundary conditions often omitted. For this manuscript, the epistemic consequence is central: over-generalization can convert context-bound results into apparently universal claims, thereby increasing the stability of dominant framings beyond what evidence warrants.

### 5.4 Automation bias and gatekeeping coupling

LLMs are entering reviewing and editorial workflows. Chen et al. (2025) found that LLM assistance reduced perceived workload and was valued for summarization and idea generation, without reliably improving review quality. Automation bias is a concern: decision makers may over-trust confident machine outputs and underweight contradictory cues. Classic experiments show that automation can increase error rates when users defer to incorrect automated recommendations (Skitka et al., 1999), and out-of-the-loop dynamics can further weaken effective human monitoring in complex systems (Merat et al., 2019). If editorial decisions depend systematically on model suggestions, acceptance and rejection can become indirectly coupled to statistical regularities of past gatekeeping, reinforcing historical hierarchies.

## 6 Epistemic governance: authority, legitimacy, and workflow safeguards

Governance claims must be analytically derived from epistemic theory rather than appended as “what should happen.” Social epistemology emphasizes that authority and legitimacy are stabilized through socially organized practices of criticism, accountability, and justified trust (Goldman, 1999; Longino, 1990). The sociology of quantification further shows how standardized indicators can convert contested judgments into seemingly objective legitimacy signals (Porter, 1995; Espeland and Stevens, 2008). Under AI-mediated workflows, epistemic agency is distributed across humans, institutions, platforms, and models. When outputs shape decisions at scale, responsibility allocation becomes structurally difficult. Constantinescu and Kaptein (2025) analyze responsibility gaps for LLM deployment in organizations and propose an interaction-based approach to responsibility distribution. The relevance here is to discipline governance implications as design constraints: if AI becomes a locus of epistemic authority in workflows, accountability and traceability must be embedded structurally rather than treated as optional norms.

Accordingly, governance in this manuscript is derived from three analytic premises:

1. **Authority without traceability increases epistemic risk**. If users cannot inspect sources, uncertainty, and boundary conditions, then trust shifts from evidence to interface fluency (Goldman, 1999).
2. **Objectivity depends on structured critical practices**. When criticism is organized and enforceable (public standards, accessible reasons, responsiveness to dissent), error-correction strengthens; when these practices are bypassed or compressed by opaque synthesis, legitimacy can detach from evidential control (Longino, 1990).
3. **Legitimacy signals become governance levers**. When metrics and standardized indicators are treated as proxies for worth, they can steer agendas and visibility; AI-mediated access can intensify this steering if retrieval and citation dynamics inherit prestige-weighted priors (Porter, 1995; Espeland and Stevens, 2008).

### 6.1 Operational safeguards as testable boundary conditions

The model predicts that AI-mediated access intensifies structural dependence *unless* safeguards are present. Examples include: (i) retrieval transparency with stable, auditable source links; (ii) explicit uncertainty and boundary-condition reporting; (iii) human oversight with documented validation checks; (iv) routine audits for citation concentration, over-generalization, and source diversity; and (v) workflow-level separation between drafting assistance and epistemic judgment (Malik and Terzidis, 2025).

These safeguards are not presented as moral preferences but as conditions that alter the causal mechanism (i.e., they constrain *f* in the mechanism sketch). They therefore appear both as governance implications and as boundary conditions in the falsifiability section.

## 7 Scope, boundary conditions, and falsifiers

### 7.1 Boundary conditions (where the mechanism weakens, fails, or reverses)

The framework is expected to weaken, fail, or partially reverse under at least four classes of conditions:

**(B1) High-traceability synthesis workflows**. Where AI is used only in retrieval-augmented settings with explicit source tracing, and outputs must pass structured validation (e.g., AI-augmented systematic reviews with documented screening, extraction, and verification), amplification and lock-in effects should decrease (Malik and Terzidis, 2025).

**(B2) Strong open-science constraints**. Fields with robust preregistration, open data, and replication cultures can constrain opportunistic metric gaming and reduce the ability of AI summaries to erase boundary conditions without detection.

**(B3) Low-prestige, high-structure corpora**. In domains where high-quality evidence is concentrated in standardized datasets, protocols, or repositories (and where retrieval is not prestige-weighted), AI-mediated discovery may increase access diversity rather than decrease it.

**(B4) Deliberate pluralism in retrieval and evaluation**. If discovery systems are explicitly designed to diversify sources (tier-aware sampling, long-tail inclusion targets, and domain-balanced retrieval) and evaluation criteria down-weight venue prestige, AI can reduce concentration and support autonomy.

### 7.2 Falsifiability and failure modes (explicit falsifiers)

The model makes empirically vulnerable claims. The following observations would count against its central mechanisms:

**(F1) No second-order concentration effect**. If AI-assisted discovery does not increase concentration on high-visibility venues/authors relative to strong baselines (with matched queries and controlled retrieval), the claim that AI acts as a structurally reinforcing second-order filter is weakened.

**(F2) No lock-in under consensus-substrate coupling**. If, across contested domains, outputs remain robustly pluralistic (high claim-cluster entropy and high attribution diversity) even when a consensus infrastructure is a major background substrate and prompts are minimally specific, consensus lock-in must be rejected or restricted.

**(F3) No epistemic consequence of metrics under AI mediation**. If metrics-based evaluation predicts behavioral adaptation but does not predict shifts in epistemic authority signals (what gets cited, taught, summarized as “state of the art”) once AI-mediated access is introduced, then the epistemic consequence pathway must be revised.

**(F4) Reverse effect under safeguards**. If audited, traceable, validation-loop workflows systematically increase long-tail inclusion and reduce prestige bias relative to baseline discovery, then the model's main risk claim should be framed as conditional on absent safeguards, not as a general property of AI-mediated access.

## 8 Redefining authorship: conceptual agency vs. technical routine

The combined effect of institutional funding, publication filters, evaluation systems, consensus infrastructures, and AI mediation raises a central question: what still defines scientific authorship?

If experimental protocols are standardized, statistical analyses are delegated to software, writing and literature search are supported by LLMs, and publication decisions depend strongly on alignment with prevailing paradigms, then the distinctive contribution of scientists cannot be the mere execution of technical routines.

Under these conditions, scientific authorship must be understood primarily as the capacity to formulate original questions, to identify neglected lines of inquiry, to develop conceptual frameworks that make hidden assumptions explicit, and to take responsibility for epistemic and ethical implications.

### 8.1 AI assistance disclosure and prompting boundary

For transparency, the author used a general-purpose LLM for language editing and structural polishing only. Prompts were restricted to instructions such as “improve clarity,” “reduce redundancy,” and “tighten argument flow,” and explicitly prohibited adding new claims, examples, or citations. All conceptual moves, definitions, empirical interpretations, and reference selections were authored and verified by the human author against the cited sources. Any AI-suggested rephrasings were accepted only after manual review to ensure that meaning, scope, and evidential commitments remained unchanged.

## 9 Discussion: testable hypotheses and minimal empirical program

Although the present article is a conceptual synthesis, the layered structural-bubble model implies empirical claims that can be tested with existing methods and publicly available corpora.

**H1 (Upstream topic selection under structural dependence)** Fields and institutions with higher dependence on agenda-bearing external funders exhibit systematically shifted topic selection toward funder-aligned framings and away from high-disruption, low-legitimacy questions.

**H2 (Visibility consolidation via publication and evaluation systems)** Controlling for transparent quality proxies, affiliation prestige, venue tier, and metric-based evaluation increase acceptance probability and downstream visibility, with measurable effects on which claims acquire epistemic authority.

**H3 (Consensus lock-in under consensus-substrate AI mediation)** When consensus-oriented substrates dominate background representations and safeguards are weak, AI-mediated access increases framing stability and reduces exposure to qualified dissent or emerging perspectives unless explicitly counter-designed.

**Design 1: AI-mediated discovery vs. baseline discovery (amplification test)** Construct matched query sets and compare (i) conventional search; (ii) retrieval-augmented AI with transparent source links; (iii) non-retrieval AI. Outcomes include source concentration, diversity, and long-tail inclusion.

**Design 2: Consensus-substrate coupling and contested-topic robustness (lock-in test)** Select topic sets differing in contestation intensity. Measure substrate stability proxies and AI framing stability and attribution diversity under conditions that include or exclude consensus-platform retrieval.

**Design 3: AI-assisted gatekeeping natural experiments (coupling test)** Use policy-change windows to test whether AI assistance shifts acceptance patterns by institution prestige, reference-list concentration, and topic novelty.

### 9.1 Conclusion

Scientific research operates inside a layered structure of dependence on funders and institutional agendas, on publication and evaluation systems, on consensus infrastructures, and increasingly on AI systems that mediate access to knowledge. These layers jointly create a structural bubble in which some questions, methods, and conclusions are structurally favored while others are systematically disfavored or rendered less visible.

Large language models do not dissolve this structure. Under weak safeguards, they can intensify it through inherited bias, citation concentration, and over-generalization (Gallegos et al., 2024; Algaba et al., 2025; Peters and Chin-Yee, 2025). Under strong workflow safeguards (traceability, validation loops, and oversight), those effects should weaken and can partially reverse (Malik and Terzidis, 2025). The proposed empirical program provides a direct route to testing these conditional claims.

## Data Availability

The original contributions presented in the study are included in the article/supplementary material, further inquiries can be directed to the corresponding author/s.
